# Supplementary material for: Homer1 Protects against Retinal Ganglion Cell Pyroptosis by Inhibiting Endoplasmic Reticulum Stress-Associated TXNIP/NLRP3 Inflammasome Activation after Middle Cerebral Artery Occlusion-Induced Retinal Ischemia
Source: Int J Mol Sci. 2023 Nov 27;24(23):16811. doi: 10.3390/ijms242316811 (PMC10706256; doi:10.3390/ijms242316811)
Supplement: Supplementary file 1 [file ijms-24-16811-s001.zip › ijms-2663607-supplementary.pdf]

Supplement materials

Materials and methods

Coimmunoprecipitation (Co-IP)

Retinal tissue is homogenized to extract total protein. For co-IP, lysates of retinal tissue were immunoprecipitated with IP buffer containing IP antibody-coupled Protein A/G PLUS-Agarose beads, and protein-protein complexes were later subjected to western blot. IgG was used as a negative control. The following antibodies were used: rabbit anti-Homer1 (ab184955; 1:60; Abcam), and rabbit anti-AMPK (#2532; 1:100; CST).

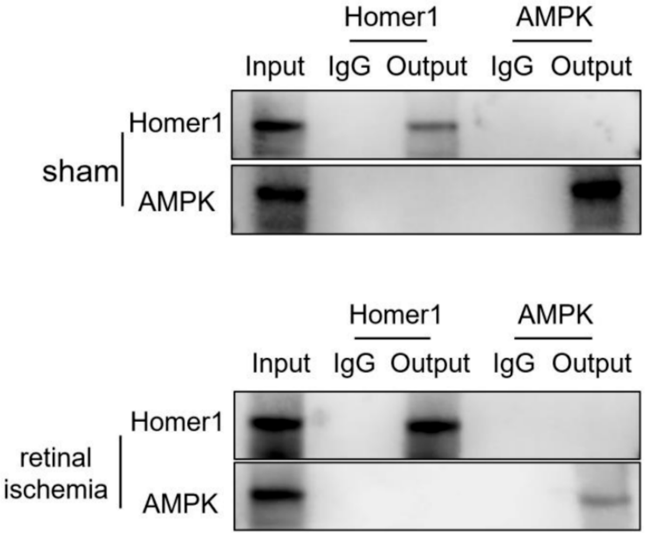

Supplement Figure S1: **There is no direct binding between Homer1 and AMPK.** Co-IP assay was conducted to detect the physical bind ability between Homer1 and AMPK in the sham and retinal ischemia group by using anti-Homer1 and AMPK antibodies.
